# Interspecific information on predation risk affects nest site choice in a passerine bird

**DOI:** 10.1186/s12862-018-1301-3

**Published:** 2018-12-04

**Authors:** Jere Tolvanen, Janne-Tuomas Seppänen, Mikko Mönkkönen, Robert L. Thomson, Hannu Ylönen, Jukka T. Forsman

**Affiliations:** 1 Department of Ecology and Genetics, University of Oulu, 90014 Oulu, Finland; 2 Nature and Game Management Trust, Degerby, Finland; 3 Open Science Centre, University of Jyvaskyla, PO Box 35, 40014 University of Jyvaskyla, Jyväskylä, Finland; 4 Department of Biological and Environmental Sciences, University of Jyvaskyla, PO Box 35, 40014 University of Jyvaskyla, Jyväskylä, Finland; 5 Section of Ecology, Department of Biology, University of Turku, 20014 Turku, Finland; 6 Percy FitzPatrick Institute of African Ornithology, DST-NRF Centre of Excellence, University of Cape Town, 7701 Rondebosch, South Africa; 7 Department of Biological and Environmental Science, University of Jyvaskyla, Konnevesi Research Station, 44300 Konnevesi, Finland; 8 Current Address: Natural Resources Institute Finland (Luke), University of Oulu, Paavo Havaksen tie 3, 90014 Oulu, Finland

**Keywords:** Social information, Nest site choice, Predation risk, Realized niche, Species coexistence, Intraspecific variation

## Abstract

**Background:**

Breeding site choice constitutes an important part of the species niche. Nest predation affects breeding site choice, and has been suggested to drive niche segregation and local coexistence of species. Interspecific social information use may, in turn, result in copying or rejection of heterospecific niche characteristics and thus affect realized niche overlap between species. We tested experimentally whether a migratory bird, the pied flycatcher *Ficedula hypoleuca*, collects information about nest predation risk from indirect cues of predators visiting nests of heterospecific birds. Furthermore, we investigated whether the migratory birds can associate such information with a specific nest site characteristic and generalize the information to their own nest site choice.

**Results:**

Our results demonstrate that flycatchers can use the fate of heterospecific nesting attempts in their own nest site choice, but do so selectively. Young flycatcher females, when making the decision quickly, associated the fate of an artificial nest with nest-site characteristics and avoided the characteristic associated with higher nest predation risk.

**Conclusions:**

Copying nest site choices of successful heterospecifics, and avoiding choices which led to failed attempts, may amplify or counter effects of nest predation on niche overlap, with important consequences for between-species niche divergence-convergence dynamics, species coexistence and predator-prey interactions.

**Electronic supplementary material:**

The online version of this article (10.1186/s12862-018-1301-3) contains supplementary material, which is available to authorized users.

## Background

The niche concept is a central tenet in the theory of species coexistence and community ecology, stating that two species cannot coexist without adequate niche differences [1–4]. One important axis of the species niche is the characteristics of offspring production site (nest, den, etc). Choice of the offspring production (breeding) site determines the available resources and threats that the animal and its offspring encounter, making it an important fitness-related decision. Resources and predation risk are also affected by the decisions of other individuals in the community, including those of other species.

Breeding site choice driven by varying nest predation pressure has been shown to be an important mechanism affecting species coexistence [5–7]. This idea is based on functional responses of nest predators to higher overall nest density in a specific microhabitat when two or more species prefer the same microhabitat [5, 7, 8]. Increased nest predation rates in each species would then select for niche divergence in microhabitat or nest site choice, and thereby facilitate local coexistence of the species [5–7]. Besides affecting species coexistence, nest predation is an important general selective force in animals [9, 10]. It usually results in complete brood loss and for short-lived species, failing even a single breeding attempt may result in zero life-time reproductive success. However, nest predation risk varies in space and time (e.g. [11–13]). Consequently, the ability of individuals to respond to cues on the relative risk of nest predation in different habitats and times should be highly adaptive.

Direct observations of often stealthy, widely ranging and quickly moving nest predators are relatively rare events for an observer, and therefore offer little information for decision-making. Encounters with nest predators may also threaten the observer itself (e.g. [14, 15]). Yet, for example, birds can clearly respond to the density of nest predators when making habitat choices [16] and offspring investment decisions [17]. A potential mechanism to estimate predator incidence without direct visual or acoustic detection is cueing on indirect signs of predators such as odour, excrements or other traces [18–22]. Behaviour, breeding site choices and success of other conspecific or heterospecific individuals may also provide information about predation risk [23–25]. A potentially profitable strategy is to follow the decisions and the resulting success (e.g. nest depredated or not) of those heterospecifics which breed a little earlier than the observer [24, 26]. Breeding attempts of such heterospecifics could provide the most up-to-date information about nest predation risk.

Heterospecific social information use is expected to be most useful between ecologically similar species [24], in this case between species that are threatened by a similar set of predators. Instances of social information use, such as heterospecific attraction and copying of behaviours (e.g. [26–30]) can result in positive fitness effects for the information user [27]. Thus social information use may favor maintaining or increasing ecological similarity between species in a community. However, ecological similarity also increases competition for shared resources (e.g. food, nest sites), favoring decreasing ecological similarity between competing species: niche divergence via character displacement is a central paradigm for species coexistence theory [1–4]. Nest predation may therefore trigger both divergence *and* convergence of realized niches, and both could conceivably be amplified by social information use. Resulting dynamics can be complex, scale-sensitive, and highly dependent on local conditions and community composition.

Here, we tested experimentally whether a migratory, cavity-nesting bird, the pied flycatcher (*Ficedula hypoleuca*), collects information about nest predation risk from indirect cues of predators visiting nests of a heterospecific resident bird, the great tit (*Parus major*). We then investigated whether the migratory bird can associate such information with a specific nest site characteristic, are able to generalize the perceived information and use it in guiding their own nest-site choice. Great tits and flycatchers are putative competitors [31, 32], yet flycatchers use tits as a source of information in many crucial decisions, such as breeding site choice and offspring investment [27, 30, 33, 34]. If pied flycatchers can associate predation risk with a particular nest-site characteristic of their putative competitors, we expect them to prefer vacant nest sites exhibiting the same characteristic as the ‘low-risk’ nest sites of heterospecifics.

## Methods

**Fig. 1:**
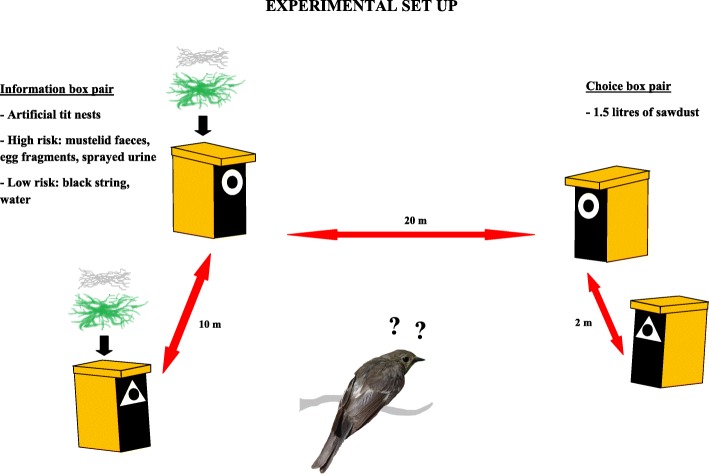
Schematic illustration of the experimental set up

We conducted the field experiment in northern Finland (N 65°, E 25°) during breeding seasons in 2013–2016. The experimental design consisted of separate nest box sites set up in habitats suitable for breeding pied flycatchers, at least one kilometre apart to improve independence. Each site included two pairs of nest boxes (see Fig. 1 for a schematic illustration of the experimental design). In one pair (the ‘information box pair’) we constructed inside both nest boxes artificial great tit nests of moss and sheep hair. We assume flycatchers perceived these artificial nests as genuine tit nests, or at least as heterospecific nests, because their own nests consist solely of plant material (wood bark, hay) and are clearly distinguishable from tit nests.

The artificial nests were randomly assigned to treatments with or without simulated nest predator visits. One of the boxes represented the ‘high-risk’ nest site, where we sprayed faeces and urine of mustelids (*Mustela nivalis nivalis*, *Mustela erminea* and *Martes martes*; abundant nest predators in the study area) dissolved in water inside and outside the nest box and on the base of the tree the nest box was attached to. The water-excrement mix was made by mixing sawdust containing mustelid urine and faeces with water and letting the urine and faeces dissolve in the water over two days (see [19] for more detailed information). Sawdust containing mustelid faeces and urine was obtained from cages of captive individuals at Konnevesi Research Station and Ranua Zoo. In addition, we put two pieces of mustelid faeces and few fragments of a common hen (*Gallus Gallus domesticus*) egg on top of the nest. The other nest box within the ‘information box pair’ was treated using only water in spraying and putting two pieces of black string (control for mustelid faeces) on top of the nest, without any egg fragments.

We then attached a neutral symbol, made of white plastic, around the entrance hole (3.2 cm diameter) of each box within the box pair; one box got a triangle and the other one a circle, diameter or side dimension 7.5 cm. Across the experimental sites we systematically randomized which symbol was associated with the ‘high-risk’ nest site (the nest with simulated predator visit) so that in half of the sites a triangle was associated with the ‘high-risk’ site and vice versa. Instead of using some naturally occurring nest-site difference, such as cavity entrance height or diameter or tree species, we purposefully used a novel, neutral characteristic pair with just one very salient but simple contrast (shape), to which birds should not have any intrinsic or learned response. Such designs isolate behavioral traits under investigation from confounding factors [33, 35] and have been successfully applied in earlier nest-site choice studies (e.g. [30, 33]). The boxes within the ‘information box pair’ were set up ca. 10 m apart in trees of same size and species.

About 20 m away from the ‘information box pair’ we set up another box pair, the ‘choice box pair’. This box pair included two vacant nest boxes ca. two meters apart, again set up in similar trees. We put 1.5 l of clean sawdust into each of the ‘choice box pair’ boxes to make these vacant boxes more attractive nesting sites than the two in the ‘information box pair’. Flycatchers strongly prefer building their nest on top of existing nest materials, such as dummy [36] or deserted (pers. obs.) tit nests, and settling in the ‘information box pair’ would yield no data on flycatcher’s ability to associate neutral symbol with nest predation risk. However, clean sawdust in the nest box is preferred even more strongly than tit nests [37].

Finally, we randomly assigned the two symbols, triangle and circle, to the ‘choice box pair’ boxes. This created a setting where two equally attractive vacant nesting sites were available immediately adjacent to each other, for flycatcher to choose between. These nest sites differed only in the type of symbol attached around the cavity entrance, while the nearby ‘information box pair’ featured association between one of the symbols and nest predation risk. The distance between the box pairs had to be relatively short to ensure that the birds settling in the ‘choice box pair’ most likely encounter the simulated information in the ‘information box pair’ before constructing their own nest. Due to the small spatial scale this experimental design is conservative: it is conceivable that birds may perceive predation risk to be uniform in the general area, i.e. equal in all nest boxes within a site, and consequently do not respond to the symbol-risk association. Finding a significant response would thus give strong support to the existence of abilities in birds to i) detect indirect cues of predation risk from observing heterospecific nesting attempts and ii) associate nesting site characteristics with that predation risk information and iii) develop preference for “safer” characteristics in their own nest-site choice.

We monitored the settlement and breeding of pied flycatchers by visiting the sites usually every second day (occasionally every third day). During each visit we refreshed the treatments within the ‘information box pair’ (repeated the spraying and ensured that faeces/strings and egg fragments continued to be observable) and checked the flycatcher nest status in the ‘choice box pair’. We recorded the emerging flycatcher nests according to a four-level classification: some nest material in the box, but box floor still visible (level 1), half nest (box floor not visible) but no cup-shape (level 2), cup-shape forming, but cup not yet completed (level 3) and ready nest for laying (cup completed; level 4). Usually the birds initially brought some nest material to both of the ‘choice’ boxes, but eventually completed the nest in only one of them. We defined the choice to have happened once we observed at least a two-level difference between the adjacent vacant nest boxes.

Pied flycatcher males usually arrive to breeding sites before females and defend a territory that may include several potential nest cavities (e.g. both nest boxes in our ‘choice box pair’), while the female builds the nest and thus presumably has more influence than the male on the nest-site choice ([14]; but see [38]). We therefore concentrated on female behavior. We approximated female arrival time as the nest initiation day, defined as the day when a level 1 nest was first observed, or as the previous day in case the nest was first observed as a further level nest. We captured females from the still active nests during incubation (two nest were abandoned earlier), determined their age as young (1-year-old) or old (at least 2 years old) based on plumage characteristics [39] and measured their tarsus length as a proxy of body size.

The value of social information is expected to show temporal degradation as the time lag between the emergence of information (behavior or success of the information source) and its application (by the information user) increases [24]. It is thus expected that individuals using social information would apply the information quickly after obtaining it. In addition, information based on social cues is often more easily available compared to information collected directly from the environment (e.g. by observing predators). Thus, individuals that base their decisions on social information are expected to make faster decisions than those who use direct personal observations to obtain information [40–42]. To take the time used to make the nest site (symbol) choice into account, we calculated a variable ‘decision time’ as the difference between the choice date (day when at least a two-level difference in the nest stage was observed) and the approximate arrival date. If social information use results in faster decision-making, we expect birds using the simulated information to have shorter decision time than those not using the information (i.e., making random symbol choice).

Statistical analyses were performed using generalized linear models with binomial error distribution in Program R (version 3.3.1; [43]). The full model included the variables female age, tarsus length, arrival time (both linear and quadratic terms), decision time, year (2013–2016) and chosen symbol (triangle or circle). In addition, we fitted two-way interactions between female age and tarsus length, arrival time and decision time, as well as two-way interactions between tarsus length and arrival time and decision time. Continuous explanatory variables were mean-centered (arrival time to year-specific means) prior to analyses. Since the analysis of the full data indicated an interaction related to female age, we repeated the analyses for old and young females separately. In these analyses the full model was the same as for the full data, but without the female age variable and its interactions.

After defining the full model, all biologically reasonable models under the full model were fitted to the data and Akaike’s information criterion [44] corrected for small sample size, AICc, was used to rank the models. To take model selection uncertainty into account we derived top model sets that included all models with ΔAICc < 6, but with the constraint that models that were more complex versions of a model with lower AICc were omitted [45]. If more than one model was included in the final top model set, we evaluated the relative support between the models using evidence ratios (ratios of model Akaike weights; [46]). We also present effect sizes with 95% confidence intervals for variables in the best supported models. Collinearities of continuous explanatory variables were estimated using Pearson correlation coefficients, but all pair-wise correlations were low (*r* < 0.29). Both full and final models within the top model sets (if included at least two explanatory variables) were also tested for overall multicollinearity using variance inflation factors (VIF), but all VIFs were acceptable (< 2.8). Also overdispersion levels of full and final models were acceptable (sum of squared Pearson residuals /residual df < 1.14).

## Results

We recorded 113 pied flycatcher nest site choices within the ‘choice box pairs’, but two nests were abandoned before we could capture the females. Therefore the data set used in the analysis included 111 nest site choices, of which 62 were made by old females and 49 by young females. Overall, flycatchers chose the symbol depicting ‘low-risk’ nest site in 62 of 111 (56%) cases; old females in 32 of 62 (52%) and young females in 30 of 49 (61%) cases. Only 15 flycatcher pairs settled on top of the simulated tit nests (seven and eight pairs on top of nests with and without simulated nest predator visit, respectively) showing that adding sawdust in the ‘choice box pairs’ was an efficient way to get the flycatchers to settle within the desired box pair.

The analysis of the full data set (*n* = 111 choices) resulted in a top model set of two models. The best supported model included the interaction between female age and decision time (Table 1), and was considerably better supported than the second, intercept-only model (ΔAICc = 4.29; evidence ratio 0.9 / 0.1 = 9.0). Data was therefore analysed separately for old and young flycatcher females. The top model set for old females only included the intercept-only model. The intercept did not differ from zero indicating random nest site choice in respect to the symbol around the entrance hole (Table 1).

**Table 1:** Parameter estimates and their 95% confidence intervals in the best-supported models of full data, old and young female data. Statistics of statistically significant variables (95% CI excluding zero) in explaining pied flycatcher nest site choices in bold

Data set	Variable	Estimate	95% CI
Full data	Intercept	0.058	−0.443 – 0.561
	Age_young	0.363	−0.443 – 1.185
	Decision time	0.037	−0.128 – 0.214
	Age_young:Decision time	**−0.474**	**−0.845 – − 0.142**
Old females	Intercept	0.065	−0.435 – 0.567
Young females	Intercept	0.523	−0.105 – 1.194
	Decision time	**−0.437**	**−0.770 – − 0.151**

**Fig. 2:**
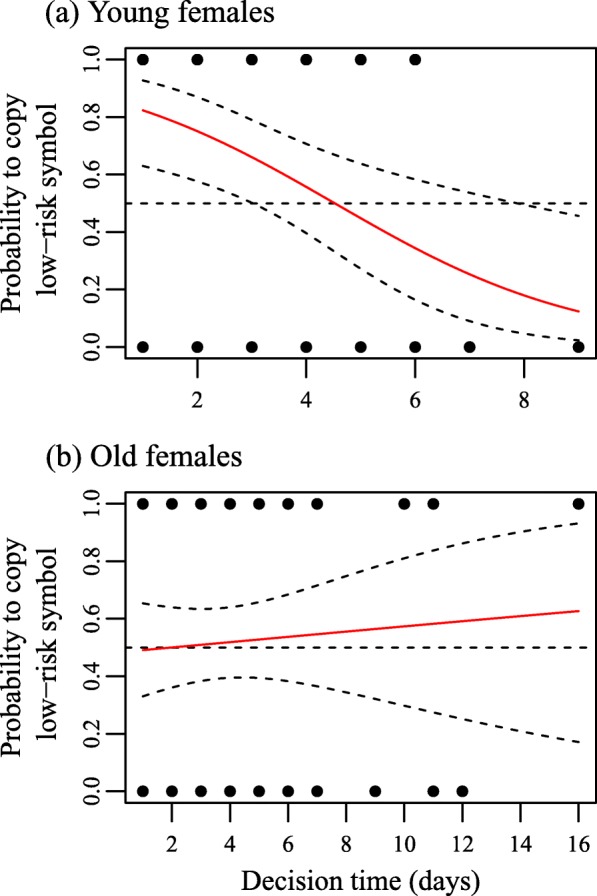
The probability of (**a**) young and (**b**) old pied flycatcher females to choose the nest site with the ‘low-risk’ symbol attached around the entrance hole in relation to the decision time. Dashed lines along the solid red line (the predicted probability) depict the 95% confidence intervals. Vertical dashed line depicts the expected probability level (0.50) under random nest site choice. Dots represent data points (each dot may include several data points). Note the different scale of the x-axis between (**a**) and (**b**)

For young females the top model set included only one model indicating strong negative effect of decision time (Table 1). We illustrate the effect of decision time on the nest site choice of young females in Fig. 2a, and for comparison illustrate the same effect on choices of old females in Fig. 2b. Both figures are based on the models including only the effect of decision time (the best model for young and the third best model for old female data). Those young females that made the nest site (symbol) choice relatively quickly, decision time < 3 days, preferred the ‘low-risk’ symbol: 18 of 23 (78%) females chose the ‘low-risk’ symbol. The preference for ‘low-risk’ symbol decreased with increasing decision time and disappeared when decision time reached 5 days or more (Fig. 2a). There are very few data points at longest decision times (*n* = 4 for decision time > 6 days), thus reversal of preference at large values should not be inferred, despite the graphical appearance. Decision time was not related to nest site choice in old females (Fig. 2b).

## Discussion

Our results demonstrate that flycatchers are able to use the fate of preceding heterospecific nesting attempts in their own nest-site choice – but do so selectively. Young flycatcher females, when making the decision quickly, associated the simulated fate of an artificial heterospecific nest with a neutral nest-site characteristic and preferred the characteristic associated with lower nest predation risk (or avoided the characteristic associated with higher risk). The choices of old females were random irrespective of the time used in decision-making.

Previous studies have shown that birds may obtain information about predators indirectly via olfaction [18, 21] or by observing excrements of mammalian predators and avoid nest sites that predators apparently had visited [19, 20, 22]. Our experiment demonstrates a more complicated response whereby birds derive information on nest predation risk from heterospecific nesting attempts (another dimension of indirect information) and associate the information to a specific nest site characteristic. This derived information may then be applied to other nest site locations with similar characteristics. For example, vegetation characteristics are often consistently associated with high and low predation risk [5–7] and by linking the observed success of others and vegetation characteristics, individuals could choose safer nest sites (or avoid risky sites) accordingly. It is likely that nesting attempts of heterospecifics and the associated microhabitat features are more easily observed than predators themselves, making their association an available information source for individuals. However, the value of such information depends on the spatio-temporal variation in predator type and behavior and it is expected to decrease with increasing distance and delay from the event that generated the information [24].

Given that nest predation poses a significant threat to breeding birds [9, 10], it is expected that individuals would collect information about predator incidence in specific nest sites or areas. Why then did not all the flycatcher females prefer the ‘low-risk’ nest sites in our experiment? Social information use strategies have been observed to exhibit age-specific variation [34, 47–50], probably due to differences in personal experience or ability and opportunity to collect information personally. Old (at least 2-year-old) females probably have prior breeding experience and may be better in collecting information personally (i.e. directly, not indirectly via other individuals). They may more readily rely on personal information, despite also having obtained the social information. For example, old females may have perceived the simulated social information but then may have observed that there are no predators present in the area (any more) and thus selected the nest site randomly in respect of the symbol. Young (1-year-old) females do not have prior breeding experience and may thus prefer to use social information. Due to the small spatial dispersion of the experimental nest boxes, more experienced old females may also have perceived the predation risk to be equal among all the boxes resulting in random nest site choice.

We observed variation also among the young females: only those that made their nest site choice decisions quickly used the simulated social information. This is consistent with the hypothesis that the value of social information decreases with increasing delay between the event generating the information and the application of that information by the observer [24]. On the other hand, social information use also enables faster information collection and thus also faster decision-making compared to using (only) personal information [40–42]. Our direct observations of flycatcher behaviour at the experimental sites showed that females may visit and therefore derive information from all the nest boxes within a site in just a few minutes. By relying on the information available in the other nests they could therefore make the nest site choice immediately after arriving to the site. Nest site choice by females that took longer to make a decision was indifferent to symbols and their associated predation treatment, and is consistent with them obtaining nest site quality information personally. Females making slower decisions may also have explored the surroundings and encountered other experimental sites with contrasting symbol-information associations (these were randomized across sites) increasing the likelihood of random choices. These results provide empirical evidence for the hypotheses of faster decision-making when relying on social information [40–42] as a response to degrading information value with time ([24]; see also [51]), mainly observed in group foraging to date [52, 53].

Cueing on nest predator presence via indirect sources through heterospecific nesting attempts considerably increases the amount of available information that can be acquired safely and quickly by breeding birds. This behavior may have important implications for realized niche overlap between species and therefore for species coexistence, community ecology, and ultimately evolution. Whereas nest predation per se selects for niche divergence in nest site choice of coexisting species [5–7], heterospecific information use in relation to nest predation risk, and more specifically copying of nest site characteristics of safe nest sites, may result in maintenance of similarity and even enhanced convergence into the ‘low-risk / predator free niche space’. On the other hand, avoidance of characteristics of risky nest sites results in divergence in the ‘high-risk niche space’. Since nest predation exerts a strong selection pressure in birds [9, 10], niche evolution should proceed towards safe nest sites in both interacting species (information source and information user). If similar characteristics define safe nest sites in both species, they should show niche convergence. Heterospecific information use could then add on the independent species-specific effects and accelerate convergence of the realized niches between the species. Information use and consequent heterospecific attraction also provide a potential explanation for the observations where competitors share similar microhabitats despite they suffer higher nest predation than when breeding alone (cf. [7]).

On the other hand, convergence in nest site characteristics via heterospecific information use would result in higher nest density in specific nest sites. If nest predators respond functionally and begin to prefer such microhabitats in searching prey, increased predation risk in this microhabitat would select for divergence in nest site niche between the two species (cf. [5–7]). As a result, the interacting species (information source and information user) could end up in fluctuating realized-niche divergenceconvergence dynamics. Pace, amplitude and spatial scale of those dynamics would depend on the functional responsiveness and other characteristics of the predator community and the prevalence of heterospecific information use among the prey species. Strong functional responsiveness of predators coupled with frequent heterospecific information use among the prey species would result in relatively rapid fluctuations occurring within few generations, while weaker responsiveness and less frequent information use could yield modest fluctuations over longer temporal scales. Spatial variation in predator and prey communities and their responsiveness and reliance on interspecific information use would create localized dynamics, and consequently diversify ecological interactions (predator-prey and information use dynamics) at larger spatial scales.

## Conclusions

This study demonstrates that birds i) can detect indirect cues of predation risk from observing heterospecifics nesting attempts and ii) can associate nesting site characteristics with that predation risk infromation and iii) can – but only conditionally do – develop preference for “safer” characteristics in their own nest site choice. Such interspecific social information use in relation to nest predation risk may affect realized niche dynamics among coexisting species with important implications for species coexistence and community dynamics. These findings also add to the accumulating evidence of between-individual variation in social information use patterns; both due to age-related differences and also due to within-age-group variation. Given the substantial potential of individual level variation in behaviour to affect key ecological and demographic processes, also including species coexistence and community ecology and evolution [54–57], we should aim to thoroughly understand the variation in information use.

## Additional file

Additional file 1: The data set supporting the article. (XLSX 16 kb)

## Abbreviations

AICc: Akaike’s information criterion corrected for small sample size
VIF: Variance inflation factor
ΔAICc: Difference in AICc units of the focal model compared to the model with lowest AICc
